# Early increased cell proliferation compensates subsequent hypoplasia of the ossicle

**DOI:** 10.3389/fcell.2025.1627730

**Published:** 2025-10-29

**Authors:** Katsushige Kawasaki, Maiko Kawasaki, Finsa Tisna Sari, Vanessa Utama, Alex Kesuma, Makoto Fukushima, Naoaki Saito, Daisuke Suda, Takehisa Kudo, Akira Fujita, Jun Nihara, Brunella Franco, Atsushi Ohazama

**Affiliations:** ^1^ Division of Oral anatomy, Faculty of Dentistry & Graduate School of Medical and Dental Sciences, Niigata University, Niigata, Japan; ^2^ Center for advanced oral science, Faculty of Dentistry & Graduate School of Medical and Dental Sciences, Niigata University, Niigata, Japan; ^3^ Divisions of Oral and Maxillofacial Surgery, Niigata University Graduate School of Medical and Dental Sciences, Niigata, Japan; ^4^ Department of Tissue Regeneration and Reconstruction, Division of Reconstructive Surgery for Oral and Maxillofacial Region, Niigata University Graduate School of Medical and Dental Sciences, Niigata, Japan; ^5^ Faculty of Dentistry & Graduate School of Medical and Dental Sciences, Division of Orthodontics, Niigata University, Niigata, Japan; ^6^ Telethon Institute of Genetics and Medicine (TIGEM), Naples, Italy; ^7^ Department of Translational Medical Sciences, Medical Genetics, Federico II University of Naples, Naples, Italy; ^8^ School for Advanced Studies (Scuola Superiore Meridionale), Genomic and Experimental Medicine Program, Naples, Italy; ^9^ Center of Excellence in Genomics and Precision Dentistry, Faculty of Dentistry, Chulalongkorn University, Bangkok, Thailand

**Keywords:** ossicle, neural crest-derived cells, Hh signaling, OFD1, IFT88, primary cilia, cell proliferation

## Abstract

Ossicles are essential structures for normal sound conduction from the external environment to the inner ear. Proper formation of the ossicles is required for normal hearing, and ossicular deformities lead to hearing loss. We identified ossicular hypoplasia in mice with mesenchymal conditional deletion of the primary cilia molecule (*Ofd1* ^*fl*^ *;Wnt1Cre* and *Ift88* ^*fl/fl*^ *;Wnt1Cre*). Hh signaling activity and cell proliferation were significantly downregulated in ossicle primordia of *Ofd1* ^*fl*^ *;Wnt1Cre* mice from E11.5. To restore Hh signaling in *Ofd1* ^*fl*^ *;Wnt1Cre* mice, we crossed *R26SmoM2* ^*fl*^ mice (a constitutively active form of Smo) with *Ofd1* ^*fl*^ *;Wnt1Cre* mice. Ossicular hypoplasia was partially rescued in *Ofd1* ^*fl*^ *;Wnt1Cre*;*R26SmoM2* ^*fl*^ mice. However, Hh signaling activity was not restored after E11.5. Instead, Hh signaling activity and cell proliferation were significantly increased in *Ofd1* ^*fl*^ *;Wnt1Cre*;*R26SmoM2* ^*fl*^ mice at E10.5, when these were not altered in *Ofd1* ^*fl*^ *;Wnt1Cre* mice. To confirm whether molecular changes at E10.5 rescue subsequent hypoplasia, SAG (agonist of Hh signaling) was applied to *Ofd1* ^*fl*^ *;Wnt1Cre* mice at E9.5. A similar rescue could be observed in *Ofd1* ^*fl*^ *;Wnt1Cre* mice with SAG application. Thus, early increased cell proliferation could compensate subsequent hypoplasia of ossicle formation. Our results may provide clues for possible future treatment in familial hearing loss due to hypoplasia of the ossicles.

## Introduction

The mammalian hearing apparatus comprises three distinct parts: the outer, middle, and inner ear. Sound waves collected by the outer ear are converted into vibrations at the tympanic membrane. These vibrations are conveyed to the chain of three ossicles (i.e., malleus, incus, and stapes) in the middle ear, which are relayed to the cochlea of the inner ear. Thus, the middle ear ossicles bridge the gap between the outer and inner ear. Therefore, the precise formation of the ossicle is required for normal hearing. Congenital ossicular deformities lead to conductive hearing loss.

The murine ossicle is initiated in the first branchial arch from embryonic day (E) E10.5, which could be identified by Sox9 expression (Ankamreddy et al., 2019). The ossicle primordium is histologically recognized as an aggregation of the mesenchyme from E13.5. The malleus and incus are clearly identified as a different structure from E14.5. The ossicular primordium could be identified by Alcian blue staining and collagen II expression, and the ossicles increase in volume and undergo ossification after E14.5. The malleus and incus are derived from the neural crest. The footplate of the stapes is of mesodermal origin, while the other parts of the stapes are formed by neural crest-derived cells (Thompson et al., 2012; Anthwal and Thompson, 2016; Amin and Tucker, 2006).

Primary cilia are immotile organelles found on the surface of almost all mammalian cells. Cilia play important roles in many biological processes, including regulating Hh signaling pathways (Bisgrove and Yost, 2006; Zaghloul and Brugmann, 2011). The primary cilium comprises a membrane-bound cylinder surrounding nine doublet microtubules that extend from the basal body. Cilia are assembled and maintained by an intraflagellar transport (IFT) system, in which multiple protein complexes move bidirectionally along the axoneme through the coordinated action of IFT motor proteins. In the IFT system, groups of protein particles are transported from the base to the tip of the cilia and from the tip to the base. The IFT particles are composed of at least 17 polypeptides, including Ift88. Perturbations in the function of primary cilia lead to a wide spectrum of human diseases, namely, ciliopathies. Congenital morphological anomalies of craniofacial organs are a major symptom of ciliopathy (Hildebrandt et al., 2011; Mill et al., 2023; Ren et al., 2023; Castiglione et al., 2014).

The *OFD1* protein can localize to the basal bodies of primary cilia. OFD1 was identified as the gene mutated in patients with oral–facial–digital syndrome type I (OFD1 syndrome), which is classified as a ciliopathy. The OFD1 protein is necessary for the formation of primary cilia (Ferrante et al., 2006). Conductive hearing loss has been reported in OFD1 patients (Yang et al., 2022; Li et al., 2023; Kyian et al., 2024). In this study, *Ofd1*-mutant mice showed hypoplasia of the ossicles, which was caused by reduced cell proliferation due to downregulation of Hh signaling from E11.5. We found that increased cell proliferation by upregulated Hh signaling at E10.5 compensated subsequent hypoplasia of the ossicles. Our findings provide hints for possible future treatment in familial hypoplasia of ossicular formation.

## Results

### Ossicle phenotypes in primary cilia molecule mutant mice

**FIGURE 1:**
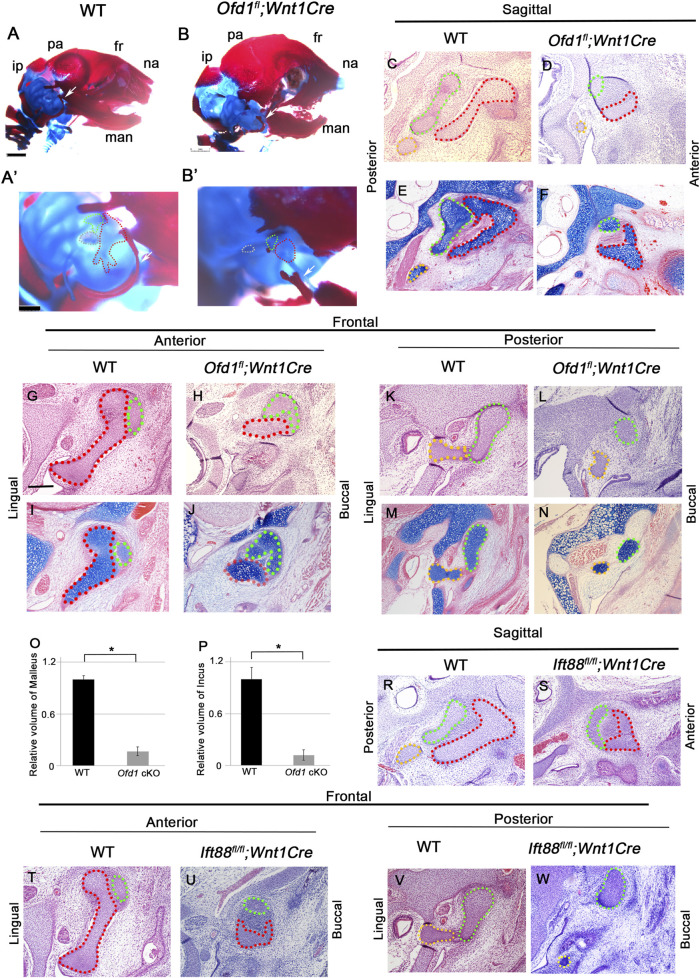
Ossicle phenotypes in *Ofd1* mutant mice (**(A)**-B′) Skeletal preparation of ossicles in wild-type (**(A)**, A′) and *Ofd1* ^*fl*^ *;Wnt1Cre* (**(B)**, B′) at E18.5. A′ and B’; high magnification of A and B, respectively. ip; interparietal bone, pa; parietal bone, fr; frontal bone, na; nasal bone, man; mandible. Malleus, incus and stapes were outlined by red, green and yellow dots, respectively. **(C-N,R-W)** Sagittal **(C-F,R,S)** and frontal **(G-N,T-W)** sections showing H&E stained **(C,D,G,H,K,L,R-W)** and alcian blue stained **(E,F,I,J,M,N)** images at ossicle region in wild-type **(C,E,G,I,K,M,R,T,V)** and *Ofd1* ^*fl*^ *;Wnt1Cre* **(D,F,H,J,L,N)** and *Ift88* ^*fl/fl*^ *;Wnt1Cre* **(S,U,W)** mice at E18.5. **(O,P)** Comparison of the volume of malleus **(O)** and incus **(P)** between wild-type (WT) and *Ofd1* ^*fl*^ *;Wnt1Cre* (*Ofd1* cKO) mice. *; P < 0.05.

To understand the role of *Ofd1* in ossicle formation, we generated and examined mice with conditional deletion of *Ofd1* in neural crest-derived cells using the *Wnt1Cre* driver. We examined only hemizygous *Ofd1* mutant mice (*Ofd1* ^*fl*^ *;Wnt1Cre*) as mutant mice since the *Ofd1* gene is located on the X-chromosome. *Ofd1* ^*fl*^ *;Wnt1Cre* mice died at birth. We examined ossicles by histological sections and skeletal preparations at E18.5. The sizes of both the malleus and incus were remarkably smaller than those in wild-type mice (n = 48/48; Figure 1; Supplementary Figure S1). The stapes in *Ofd1* ^*fl*^ *;Wnt1Cre* mice was slightly smaller than that in wild-type mice. To understand whether ossicular phenotypes in *Ofd*1-mutant mice were caused by disruption of primary cilium function, we also generated mice with mesenchymal conditional deletion of another primary cilia molecule, *Ift88*, using *Wnt1Cre* (*Ift88* ^*fl/fl*^ *;Wnt1Cre*). *Ift88* ^*fl/fl*^ *;Wnt1Cre* mice died at birth and showed similar ossicle phenotypes to those in *Ofd1* ^*fl*^ *;Wnt1Cre* mice (Figures 1R–W; Supplementary Figure S2). Thus, proper primary cilia function is essential for ossicle formation.

### Molecular changes in ossicle formation at the early stage

**FIGURE 2:**
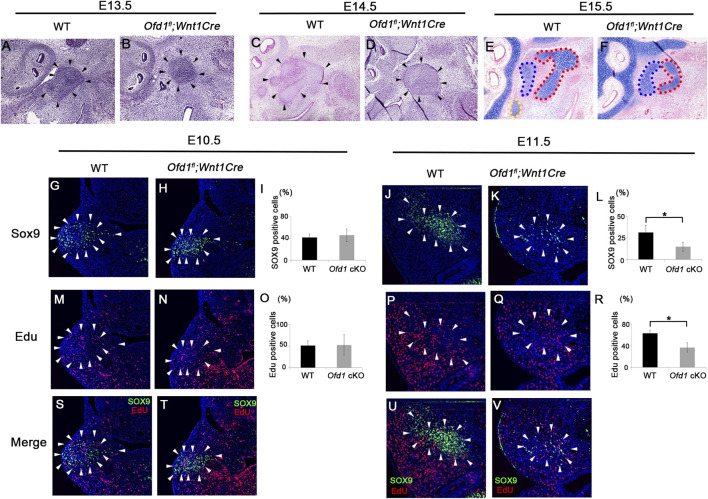
Cell proliferation and ossicle primordia **(A–F)** Sagittal **(A–F)**, colonal **(G,H,M,N,S,T)** and frontal **(J,K,P,Q,U,V)** sections showing histology **(A–D)**, alcian blue stained images **(E,F)**, and Sox9 **(G–K)**, EdU **(M,N,P,Q)** and merge **(S,T,U,V)** immunohistochemistry at E10.5 **(G,H,M,N,S,T)**, E11.5 **(J,K,P,Q,U,V)**, E13.5 **(A,B)**, E14.5 **(C,D)** and E15.5 **(E,F)**. **(I,L,O,R)** Comparison of the number of Sox9 **(I,L)** and EdU **(O,R)**-positive cells between wild-type (WT) and *Ofd1* ^*fl*^ *;Wnt1Cr* (*Ofd1* cKO) mice at E10.5 **(I,O)** and E11.5 **(L,R)**. *; P < 0.05. Arrowheads indicating Sox9 expression domain **(G,H,J,K,M,N,P,Q,S,T,U,V)**.

The ossicle primordium in *Ofd1* ^*fl*^ *;Wnt1Cre* mice was found to be smaller than that in wild-type mice at E13.5–E15.5 (Figures 2A–F). Although the ossicle primordium could not be histologically recognized at earlier stages, it could be identified as Sox9 expression at E10.5–E12.5 (Ankamreddy et al., 2019). The number of Sox9-positive cells was remarkably reduced in *Ofd1* ^*fl*^ *;Wnt1Cre* mice at E11.5, but not at E10.5 (Figures 2G–L). Reduction of Sox9-positive cells in *Ofd1* mutant mice was also observed at E12.5 (Supplementary Figure S3). We then examined cell proliferation and apoptosis within the Sox9 expression domain. Cell proliferation was significantly decreased in *Ofd1*-mutant mice at E11.5, while it showed no significant difference at E10.5 (Figures 2M–V). There were no remarkable differences in apoptosis between wild-type and *Ofd1* ^*fl*^ *;Wnt1Cre* mice at either E10.5 or E11.5 (Supplementary Figure S4). Thus, the hypoplasia of ossicular formation was likely due to reduced cell proliferation from E11.5.

**FIGURE 3:**
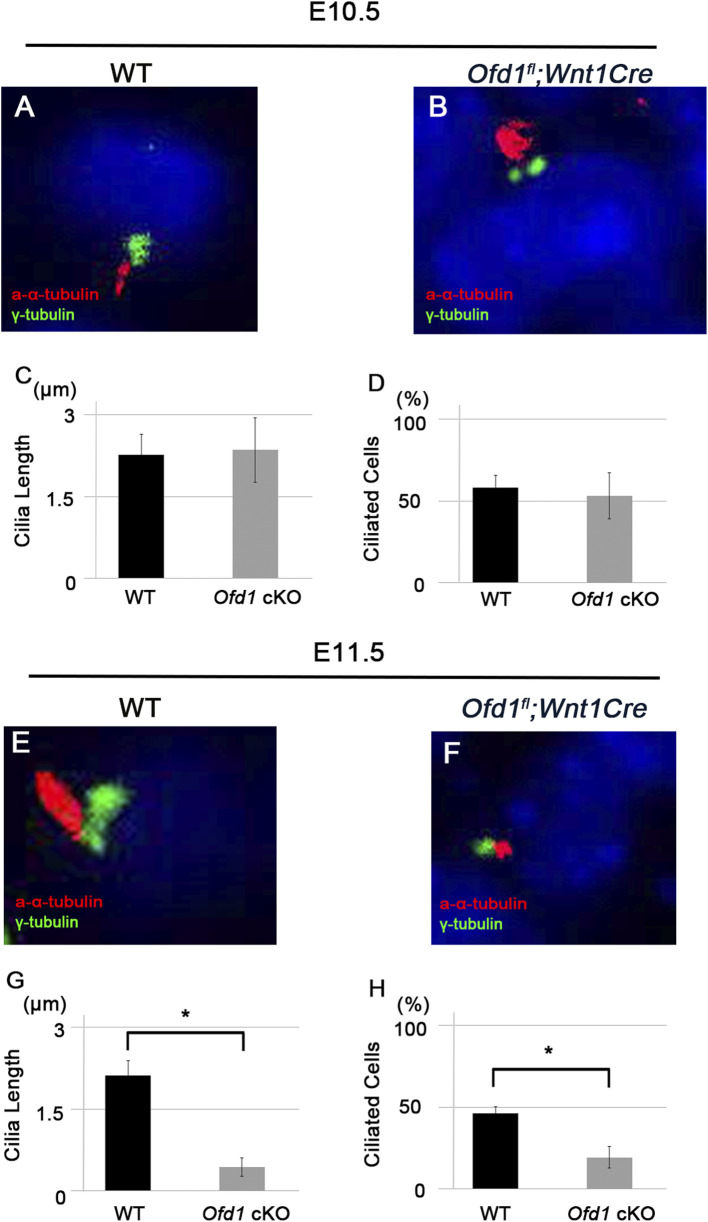
Ciliogenesis in ossicle formation **(A,B,E,F)** Coronal **(A,B)** and frontal **(E,F)** sections showing acetylated α-tubulin and γ-tubulin immunohistochemistry in wild-type **(A,E)** and *Ofd1* ^*fl*^ *;Wnt1Cre* **(B,F)** mice at E10.5 **(A,B)** and E11.5 **(E,F)**. **(C,D,G,H)** Comparison of the length of primary cilia **(C,G)** and the percentage of ciliated cells **(D,H)** between wild-type (WT) and *Ofd1* ^*fl*^ *;Wnt1Cre* (*Ofd1* cKO) mice. *; P < 0.05.

*Ofd1* deletion resulted in the lack of primary cilia in the node region (Ferrante et al., 2006). To understand whether primary cilia formation was disrupted in the ossicular region of *Ofd1* ^*fl*^ *;Wnt1Cre* mice, we performed double immunohistochemistry of acetylated α-tubulin (marker of the ciliary axoneme microtubules) and γ-tubulin (marker of the basal body). Primary cilia formation was disrupted in *Ofd1* ^*fl*^ *;Wnt1Cre* mice at E11.5, but not at E10.5 (Figure 3).

**FIGURE 4:**
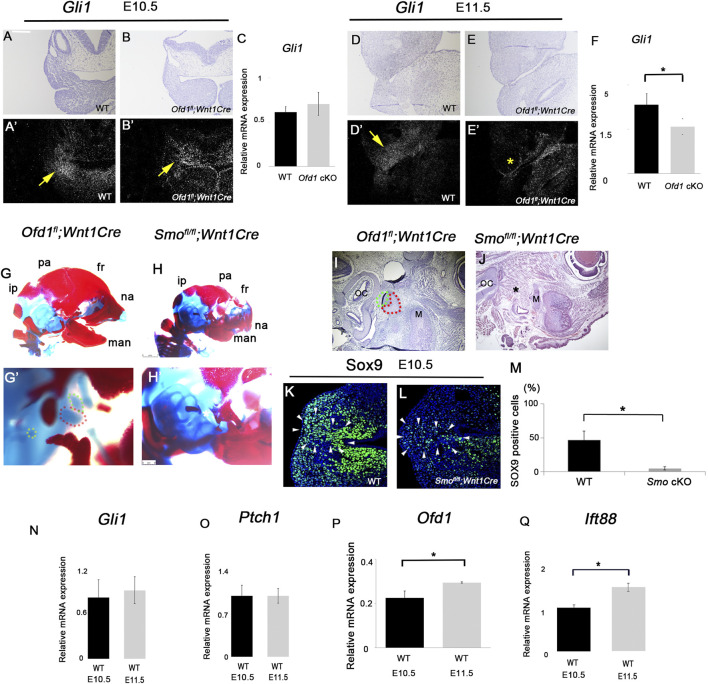
Hh signaling in ossicle formation (**(A)**-B′, (**(D)**-E′) Coronal (**(A)**-B′) and frontal (**(D)**-E′) sections showing *in situ* hybridization of *Gli1* in wild-type (**(A)**, A′, **(D)**, D′) and *Ofd1* ^*fl*^ *;Wnt1Cre* (**(B)**, B′, **(E)**, E′) at E10.5 (**(A)**-B′) and E11.5 (**(D)**-E′). **(A,B,D,E)** bright field. A′, B′, D′, E’; dark field of **(A,B,D,E)**, respectively. **(C,F)** qPCR results of *Gli1* between wild-type (WT) and *Ofd1* ^*fl*^ *;Wnt1Cr* (*Ofd1* cKO) mice at E10.5 **(C)** and E11.5 **(F)**. (**(G)**-H′) Skeletal preparation of ossicles in wild-type (**(G)**, G′) and *Smo* ^*f/fll*^ *;Wnt1Cre* (**(H)**, H′) at E18.5. ip; interparietal bone, pa; parietal bone, fr; frontal bone, na; nasal bone, man; mandible. Malleus, incus and stapes were outlined by red, green and yellow dots, respectively. *: ossicle presumptive region. G′ and H’; high magnification of **(G,H)**, respectively. **(I,J)** Sagittal sections showing histological images in wild-type **(I)** and *Ofd1* ^*fl*^ *;Wnt1Cre* **(J)** mice. Malleus and incus were outlined by red, and green dots, respectively. **(K,L)** Coronal section showing Sox9 immunohistochemistry in wild-type **(K)** and *Ofd1* ^*fl*^ *;Wnt1Cre* **(L)** mice at E10.5. Arrowheads indicating Sox9 expression domain **(K,L)**. **(M)** Comparison of the number of Sox9-positive cells between wild-type (WT) and *Smo* ^*f/fll*^ *;Wnt1Cre* (*Smo* cKO) mice. **(N–Q)** qPCR results of *Gli1* **(N)**, *Ptch1* **(O)**, *Ofd1* **(P)** and *Ift88* **(Q)** between E10.5 and E11.5 in wild-type mice (WT). *; P < 0.05.

Primary cilia are involved in Hh signaling (Mill et al., 2023). Therefore, we examined Hh signaling activity by *in situ* hybridization and qPCR analysis at these early stages. *Gli1* is a readout of Hh signaling activity, and *Ptch1* is also a major mediator of Hh signaling. In common with cell proliferation and Sox9 expression, we found that the expressions of both *Gli1* and *Ptch1* were remarkably reduced at E11.5, whereas these showed no significant differences at E10.5 (Figures 4A–F; Supplementary Figure S5A–F). Downregulation of Hh signaling in the *Ofd1* mutant was also found at E12.5 (Supplementary Figure S5G–L).

To confirm the role of Hh signaling in ossicular formation, we examined mice with conditional deletion of *Smo* (essential molecule for Hh signaling activity) in neural crest-derived cells using *Wnt1Cre* (*Smo* ^*fl/fl*^ *;Wnt1Cre* mice). Unlike *Ofd1* ^*fl*^ *;Wnt1Cre* mice with ossicular hypoplasia, the ossicles were completely absent in *Smo* ^*fl/fl*^ *;Wnt1Cre* mice (Figures 4G–J; Ankamreddy et al., 2019). Sox9 expression was significantly reduced in *Smo* ^*fl/fl*^ *;Wnt1Cre* mice at E10.5, whereas it showed no change in *Ofd1* ^*fl*^ *;Wnt1Cre* mice (Figures 4K–M), suggesting that ossicle initiation does not occur in *Smo* ^*fl/fl*^ *;Wnt1Cre* mice at E10.5, whereas it does occur in *Ofd1* ^*fl*^ *;Wnt1Cre* mice at the stage. Thus, Hh signaling at E10.5 is required for ossicle initiation, which was not impaired in *Ofd1* ^*fl*^ *;Wnt1Cre* mice. It is possible that no changes in Hh signaling in *Ofd1* ^*fl*^ *;Wnt1Cre* mice at E10.5 are caused by the failure of *Ofd1* deletion at the stage, although *Wnt1Cre* is known to be activated from E8.5 (Zalc et al., 2021). To examine the possibility, we examined *Ofd1* expression in the ossicle region of *Ofd1*-mutant mice. *Ofd1* expression was slightly observed in *Ofd1* ^*fl*^ *;Wnt1Cre* mice since the RNA sample for qPCR analysis contained mRNA from mesoderm-derived cells. However, *Ofd1* expression was significantly reduced in *Ofd1* ^*fl*^ *;Wnt1Cre* mice both at E10.5 and E11.5, indicating that *Ofd1* was successfully deleted in neural crest-derived cells of *Ofd1* ^*fl*^ *;Wnt1Cre* mice (Supplementary Figure S6). We compared Hh signaling activity in wild-type mice between E10.5 and E11.5. However, no significant difference in Hh signaling activity was observed between E10.5 and E11.5 (Figures 4N,O). It is also conceivable that Ofd1 or Ift88 plays a lesser role at E10.5 compared to that at E11.5. To examine the possibility, we compared *Ofd1* and *Ift88* expressions in wild-type mice between E10.5 and E11.5 and found that the expression levels of *Ofd1* and *Ift88* at E10.5 were significantly lower than those at E11.5 in wild-type mice (Figures 4P,Q). *Ofd1* and *Ift88* were likely dispensable for Hh signaling at E10.5, when ossicles initiated. Similar findings have been reported using *Prx1Cre* in *Ofd1*-mutant limbs (Bimonte et al., 2011). Similar to *Ofd1* ^*fl*^ *;Wnt1Cre* mice, Hh signaling was retained in *Ofd*1 mutant limbs at E10.5, although *Prx1Cre* was activated from E9.5. Reduction in Hh signaling was found in *Ofd1* limb primordia from E11.5. Next, we examined the cilia length and the percentage of ciliated cells between E10.5 and E11.5 in wild-type mice. There was no significant difference in these parameters, suggesting that the differences in *Ofd1* and *Ift88* expressions in wild-type mice between E10.5 and E11.5 are not related to the strength of ciliogenesis (Supplementary Figure S7).

### Rescue of ossicle deformities

**FIGURE 5:**
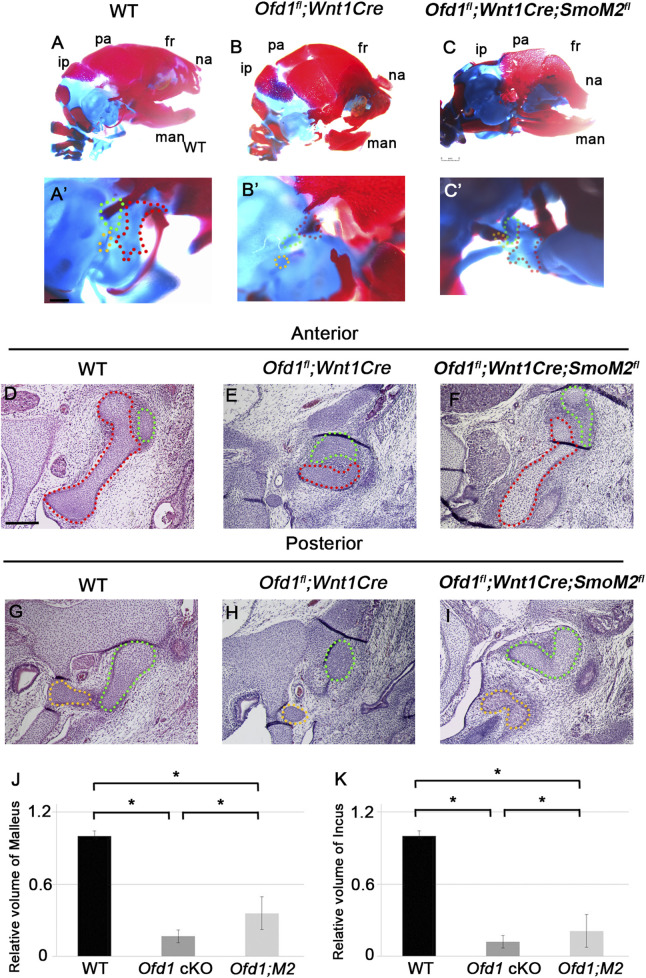
Rescue experiment in *Ofd1* mutants (**(A)**-C′) Skeletal preparation of ossicles in wild-type (**(A)**, A′), *Ofd1* ^*fl*^ *;Wnt1Cre* (**(B)**, B′) and *Ofd1* ^*fl*^ *;Wnt1Cre;R26SmoM2* ^*fl*^ (**(C)**, C′) mice at E18.5. A′, B′ and C’; high magnification of **(A–C)**, respectively. ip; interparietal bone, pa; parietal bone, fr; frontal bone, na; nasal bone, man; mandible. Malleus, incus and stapes were outlined by red, green and yellow dots, respectively. **(D–I)** Frontal sections showing histological images in wild-type **(D,G)**, *Ofd1* ^*fl*^ *;Wnt1Cre* **(E,H)** and *Ofd1* ^*fl*^ *;Wnt1Cre;R26SmoM2* ^*fl*^ **(F,I)** mice. **(J,K)** Comparison of the volume of malleus **(J)** and incus **(K)** between wild-type (WT), *Ofd1* ^*fl*^ *;Wnt1Cre* (*Ofd1* cKO) and *Ofd1* ^*fl*^ *;Wnt1Cre;R26SmoM2* ^*fl*^ (*Ofd1;M2*) mice. *; P < 0.05.

To further confirm whether ossicle hypoplasia in *Ofd1*-mutant mice was caused by the downregulation of Hh signaling, the rescue experiment was performed using *R26SmoM2* ^*fl*^ mice. Overactivation of Hh signaling was achieved by constitutive *Smo* expression in *R26SmoM2* ^*fl*^ mice, when *R26SmoM2* ^*fl*^ mice were crossed with mice with *Cre* recombinase. We crossed *Ofd1* ^*fl*^ *;Wnt1Cre* mice with *R26SmoM2* ^*fl*^ mice (*Ofd1* ^*fl*^ *;Wnt1Cre;SmoM2* ^*fl*^)*.* Ossicle phenotypes found in *Ofd1* ^*fl*^ *;Wnt1Cre* mice were partially rescued in *Ofd1* ^*fl*^ *;Wnt1Cre;SmoM2* ^*fl*^ mice (n = 12/12; Figure 5; Supplementary Figure S8).

**FIGURE 6:**
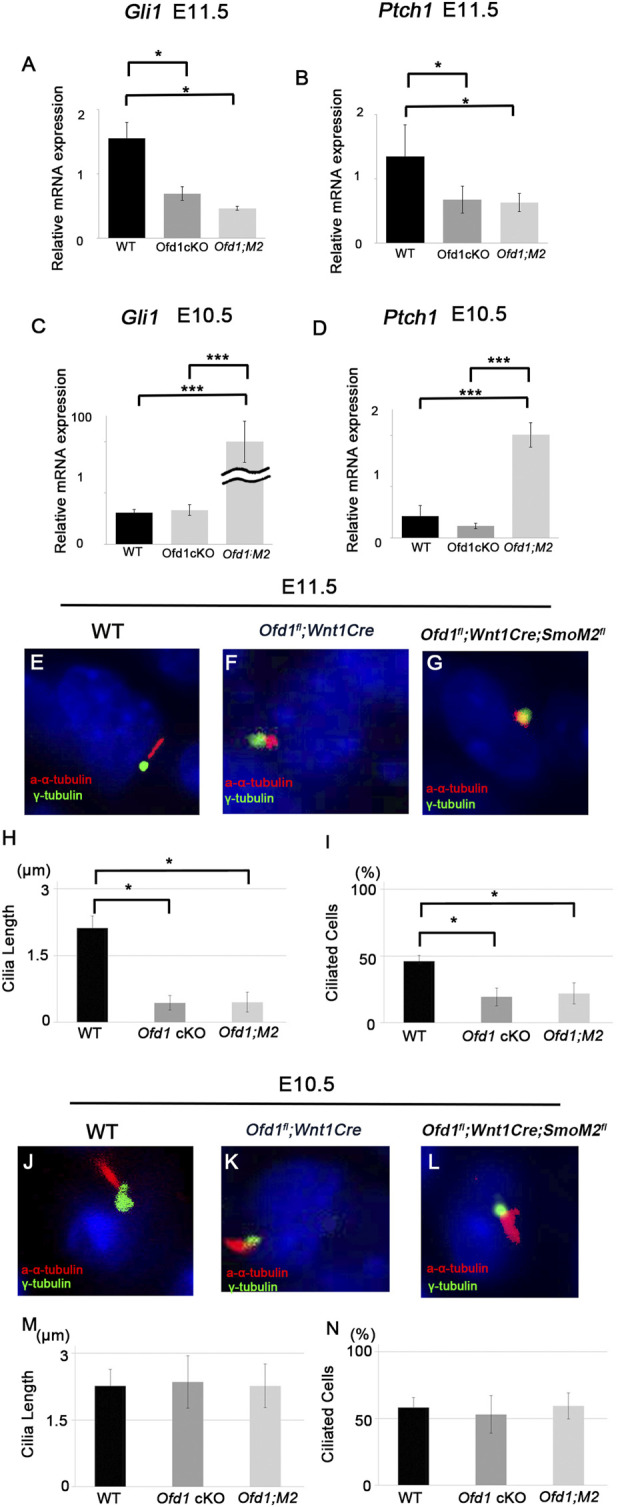
Hh signaling in *Ofd1* mutant mice **(A–D)** qPCR results of *Gli1* **(A,C)** and *Pcth1* **(B,D)** between wild-type (WT), *Ofd1* ^*fl*^ *;Wnt1Cr* (*Ofd1* cKO) and *Ofd1* ^*fl*^ *;Wnt1Cre;R26SmoM2* ^*fl*^ (*Ofd1;M2*) mice at E10.5 **(C,D)** and E11.5 **(A,B)**. *; P < 0.05, ***; P < 0.005. (E-G, J-L) Coronal **(E–G)** and frontal **(J–L)** sections showing acetylated α-tubulin and γ-tubulin immunohistochemistry in wild-type **(E,J)**, *Ofd1* ^*fl*^ *;Wnt1Cre* **(F,K)** and *Ofd1* ^*fl*^ *;Wnt1Cre;R26SmoM2* ^*fl*^ **(G,L)** mice at E10.5 **(J–L)** and E11.5 **(E–G)**. **(H,I,M,N)** Comparison of the length of primary cilia **(H,M)** and the percentage of ciliated cells **(I,N)** between wild-type (WT), *Ofd1* ^*fl*^ *;Wnt1Cre* (*Ofd1* cKO) and *Ofd1* ^*fl*^ *;Wnt1Cre;R26SmoM2* ^*fl*^ (*Ofd1;M2*) mice at E10.5 **(M,N)** and E11.5 **(H,I)**. *; P < 0.05.

However, Hh signaling was not restored inmice *Ofd1* ^*fl*^ *;Wnt1Cre;SmoM2* ^*fl*^ mice at E11.5, when ossicular deformities were recognized in *Ofd1* ^*fl*^ *;Wnt1Cre* mice (Figures 6A,B). On the other hand, Hh signaling was upregulated in *Ofd1* ^*fl*^ *;Wnt1Cre;SmoM2* ^*fl*^ mice at E10.5 in comparison with those in wild-type and *Ofd1* ^*fl*^ *;Wnt1Cre* (Figures 6C,D).

Next, we examined primary cilia formation. Primary cilia formation was disrupted in *Ofd1* ^*fl*^ *;Wnt1Cre* mice at E11.5, which was not restored in *Ofd1* ^*fl*^ *;Wnt1Cre;SmoM2* ^*fl*^ mice (Figures 6E–I). Unlike E11.5, primary cilia formation showed no changes in *Ofd1* ^*fl*^ *;Wnt1Cre;SmoM2* ^*fl*^ mice at E10.5 (Figures 6J–N). Thus, partial rescue of ossicle phenotypes was not due to changes in primary cilia in *Ofd1* ^*fl*^ *;Wnt1Cre;SmoM2* ^*fl*^ mice. Similar to Hh signaling activity, it has been shown that *Ofd1* ^*fl*^;*Prx1Cre* mice also exhibited the lack of primary cilia at E11.5, but not at E10.5 (Bimonte et al., 2011). It has been shown that non-canonical Hh signaling is often activated, when primary cilia are absent (Jenkins, 2009). However, no changes in non-canonical Hh signaling-related molecules could be observed in *Ofd1*-mutant mice at E11.5, when primary cilia were absent (Supplementary Figure S9). These indicated that partial rescue of ossicle phenotypes found in *Ofd1* ^*fl*^ *;Wnt1Cre;SmoM2* ^*fl*^ mice did not occur by the restoration of Hh signaling at E11.5, when ossicle deformities were recognized in *Ofd1* ^*fl*^ *;Wnt1Cre* mice.

**FIGURE 7:**
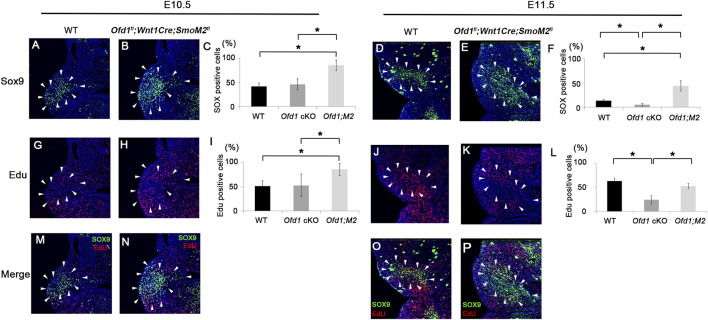
Ossicle primordia and cell proliferation in *Ofd1* ^*fl*^ *;Wnt1Cre;R26SmoM2* ^*fl*^ mice **(A,B,D,E,G,H,J,K,M-P)** Coronal **(A,B,G,H,M,N)** and frontal **(D,E,J,K,O,P)** sections showing Sox9 **(A,B,D,E)**, EdU **(G,H,J,K)** and merge **(M,N,O,P)** immunohistochemistry in wild-type **(A,D,G,J,M,O)** and *Ofd1* ^*fl*^ *;Wnt1Cre;R26SmoM2* ^*fl*^ **(B,E,H,K,N,P)** mice at E10.5 **(A,B,G,H,M,N)** and E11.5 **(D,E,J,K,O,P)**. **(C,I,F,L)** Comparison of the number of Sox9 **(C,I)** and EdU **(F,L)**-positive cells between wild-type (WT), *Ofd1* ^*fl*^ *;Wnt1Cr* (*Ofd1* cKO) and *Ofd1* ^*fl*^ *;Wnt1Cre;R26SmoM2* ^*fl*^ (*Ofd1;M2*) mice at E10.5 **(C,I)** and E11.5 **(F,L)**. *; P < 0.05. Arrowheads indicating Sox9 expression domain **(A,B,D,E,G,H,J,K,M,N,O,P)**.

We then examined ossicle primordia by Sox9 expression at both E10.5 and E11.5 in *Ofd1* ^*fl*^ *;Wnt1Cre;SmoM2* ^*fl*^ mice. The number of Sox9-expressing cells was significantly increased at E10.5 in *Ofd1* ^*fl*^ *;Wnt1Cre;SmoM2* ^*fl*^ mice compared to that in either wild-type or *Ofd1* ^*fl*^ *;Wnt1Cre* mice (Figures 7A–C). At E11.5, when ossicle deformities occurred in *Ofd1* ^*fl*^ *;Wnt1Cre* mice, the number of Sox9-expressing cells in *Ofd1* ^*fl*^ *;Wnt1Cre;SmoM2* ^*fl*^ mice was still larger than that in either wild-type or *Ofd1* ^*fl*^ *;Wnt1Cre* mice (Figures 7D–F). We then examined cell proliferation within the Sox9 expression domain. The number of cells with cell proliferation was significantly increased at E10.5 in *Ofd1* ^*fl*^ *;Wnt1Cre;SmoM2* ^*fl*^ mice compared to either wild-type or *Ofd1* ^*fl*^ *;Wnt1Cre* mice (Figures 7G–I,M,N). At E11.5, cell proliferation activity was reduced in *Ofd1* ^*fl*^ *;Wnt1Cre;SmoM2* ^*fl*^ mice, reaching a level similar to that in wild-type mice (Figures 7J–L,O,P). Cell proliferation activity in *Ofd1* ^*fl*^ *;Wnt1Cre;SmoM2* ^*fl*^ mice at E11.5 was still larger than that in *Ofd1* ^*fl*^ *;Wnt1Cre* mice (Figure 7L). At E12.5, ossicle primordia in *Ofd1* ^*fl*^ *;Wnt1Cre;SmoM2* ^*fl*^ mice were still larger than those in *Ofd1* ^*fl*^ *;Wnt1Cre* mice, whereas they were similar in size to those in wild-type mice (Supplementary Figures S10A–C). However, the number of cells with cell proliferation activity was further reduced, which was similar to that in *Ofd1* ^*fl*^ *;Wnt1Cre* mice at the stage (Supplementary Figures S10D–F). It is possible that less volume of the ossicle primordium was dependent on the size of the pharyngeal arch. We counted the number of all cells in the first pharyngeal arch. In all wild-type, *Ofd1* ^*fl*^ *;Wnt1Cre*, and *Ofd1* ^*fl*^ *;Wnt1Cre;SmoM2* ^*fl*^ mice, the number of cells in the first pharyngeal arch was significantly increased at E11.5 in comparison with those at E10.5. However, there were no significant differences between wild-type, *Ofd1* ^*fl*^ *;Wnt1Cre*, and *Ofd1* ^*fl*^ *;Wnt1Cre;SmoM* ^*fl*^ mice at either E10.5 or E11.5 (Supplementary Figure S11). Thus, changes in the size of the ossicular primordium were not due to changes in the size of the pharyngeal arch between wild-type, *Ofd1* ^*fl*^ *;Wnt1Cre*, and *Ofd1* ^*fl*^ *;Wnt1Cre;SmoM2* ^*fl*^ mice.

**FIGURE 8:**
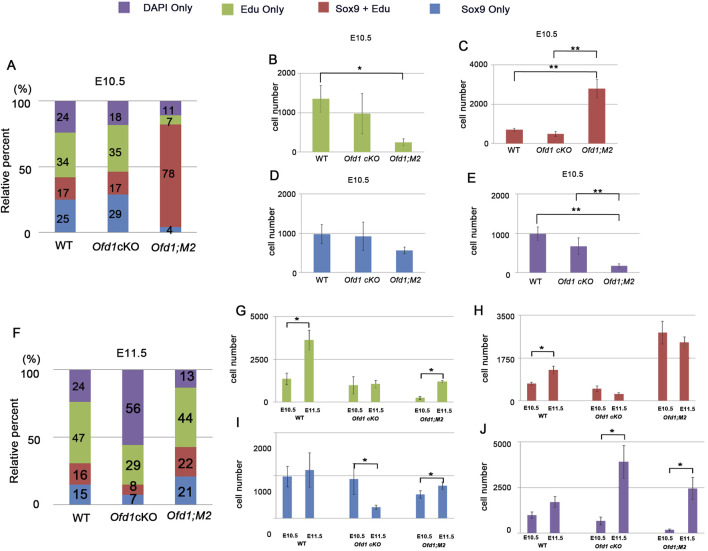
Comparison of cell proliferation and ossicle primordia **(A,F)** Cumulative bar graph showing cell composition of DAPI, EdU and Sox9-positive cells in presumptive ossicle region of wild-type (WT), *Ofd1* ^*fl*^ *;Wnt1Cre* (*Ofd1* cKO) and *Ofd1* ^*fl*^ *;Wnt1Cre;R26SmoM2* ^*fl*^ (*Ofd1;M2*) mice at E10.5 **(A)** and E11.5 **(F)**. **(B–E)** Comparison of only EdU **(B)**, EdU and Sox9 **(C)**, only Sox9 **(D)** and only EdU **(E)** between wild-type (WT), *Ofd1* ^*fl*^ *;Wnt1Cre* (*Ofd1* cKO) and *Ofd1* ^*fl*^ *;Wnt1Cre;R26SmoM2* ^*fl*^ (*Ofd1;M2*) mice at E10.5. **(G–J)** Comparison of only EdU **(G)**, EdU and Sox9 **(H)**, only Sox9 **(I)** and only EdU **(J)** in wild-type (WT), *Ofd1* ^*fl*^ *;Wnt1Cre* (*Ofd1* cKO) and *Ofd1* ^*fl*^ *;Wnt1Cre;R26SmoM2* ^*fl*^ (*Ofd1;M2*) mice between E10.5 and E11.5.

Then, we examined which type of cells was increased or decreased at E10.5 and E11.5. In wild-type mice at E10.5, 51% of mesenchymal cells showed cell proliferation activity as non-ossicular primordial cells (34%; only EdU-positive cells) and ossicular primordial cells (17%; both EdU- and Sox9-positive cells) (Figure 8A). Forty-nine percent of mesenchymal cells showed no cell proliferation activity as non-ossicular primordial cells (24%; only DAPI-positive cells) and ossicular primordial cells (25%; only Sox9-positive cells) in wild-type mice at E10.5 (Figure 8A). There were no significant differences in the number of each type of cells between wild-type and *Ofd1* ^*fl*^ *;Wnt1Cre* mice at this stage (Figures 8B–E). At E11.5, the number of cells with cell proliferation as non-ossicular primordial cells (47%; only EdU-positive cells) and ossicular primordial cells (16%; both EdU- and Sox9-positive cells) was significantly increased in wild-type mice; however, these cells were not increased in *Ofd1* ^*fl*^ *;Wnt1Cre* mice (Figures 8F–H). In *Ofd1* ^*fl*^ *;Wnt1Cre* mice at E11.5, more than half mesenchymal cells were identified as non-ossicular primordial cells without cell proliferation (Figure 8F, 56%; only DAPI-positive cells). In addition, the number of ossicular primordial cells without cell proliferation (only Sox9-positive cells) was significantly reduced in *Ofd1* ^*fl*^ *;Wnt1Cre* mice at this stage (Figure 8I). Thus, in *Ofd1* ^*fl*^ *;Wnt1Cre* mice, many mesenchymal cells lost identity as ossicular primordial cells at E11.5. Instead, in *Ofd1* ^*fl*^ *;Wnt1Cre;SmoM2* ^*fl*^ mice at E10.5, most of mesenchymal cells were identified as ossicular primordial cells with cell proliferation (Figure 8A, 78%; both EdU- and Sox9-positive cells), and they retained their status at E11.5 (Figure 8H). Other types of cells were also significantly increased in *Ofd1* ^*fl*^ *;Wnt1Cre;SmoM2* ^*fl*^ mice (Figures 8G,I,J).

**FIGURE 9:**
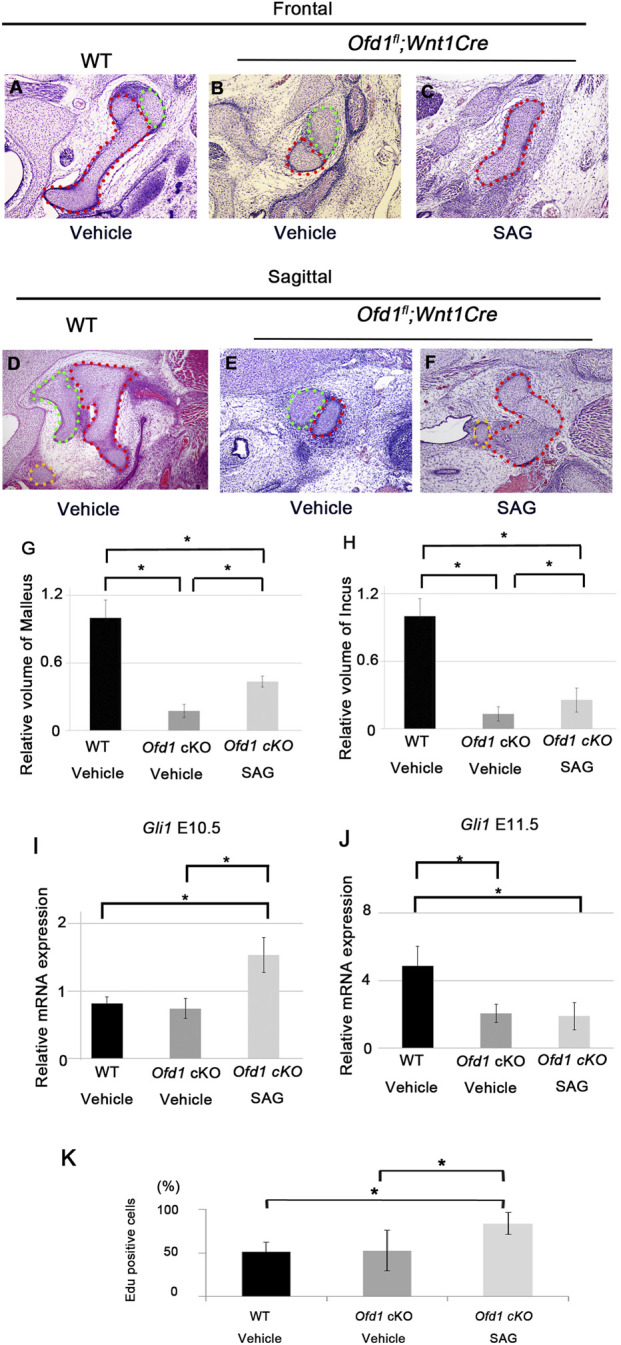
Ossicle primordia by SAG application Frontal **(A–C)** and sagittal **(D–F)** sections showing ossicle primordia in wild-type mice **(A,D)**, *Ofd1* ^*fl*^ *;Wnt1Cre* mice with vehicle **(B,E)** and *Ofd1* ^*fl*^ *;Wnt1Cre* with SAG **(C,F)**. Malleus, incus and stapes were outlined by red, green and yellow dots, respectively. **(G,H)** Comparison of the volume of malleus **(G)** and incus **(H)** between vehicle treated wild-type (WT Vehicle), Vehicle treated *Ofd1* ^*fl*^ *;Wnt1Cre* (*Ofd1* cKO Vehicle) and SAG treated *Ofd1* ^*fl*^ *;Wnt1Cre* (*Ofd1* cKO SAG) mice. **(I,J)** qPCR results of *Gli1* between vehicle treated wild-type (WT Vehicle), Vehicle treated *Ofd1* ^*fl*^ *;Wnt1Cre* (*Ofd1* cKO Vehicle) and SAG treated *Ofd1* ^*fl*^ *;Wnt1Cre* (*Ofd1* cKO SAG) mice at E10.5 **(I)** and E11.5 **(J)**. **(K)** Comparison of EdU positive cells between vehicle treated wild-type (WT Vehicle), Vehicle treated *Ofd1* ^*fl*^ *;Wnt1Cre* (*Ofd1* cKO Vehicle) and SAG treated *Ofd1* ^*fl*^ *;Wnt1Cre* (*Ofd1* cKO SAG) mice at E10.5. *; P < 0.05.

To confirm whether increased cell proliferative activity at E10.5 due to upregulated Hh signaling rescued subsequent hypoplasia of ossicle formation in *Ofd1* ^*fl*^ *;Wnt1Cre;SmoM2* ^*fl*^ mice, we injected SAG (Hh signaling agonist) into pregnant mice carrying *Ofd1* ^*fl*^ *;Wnt1Cre* mice *in utero* at E9.5 since changes in cell proliferation were already found at E10.5 in *Ofd1* ^*fl*^ *;Wnt1Cre;SmoM2* ^*fl*^ mice. Partial rescues were observed in *Ofd1* ^*fl*^ *;Wnt1Cre* mice with SAG application (Figures 9A–H; Supplementary Figure S12A–E; n = 3/6). As in *Ofd1* ^*fl*^ *;Wnt1Cre;SmoM2* ^*fl*^ mice, SAG treatment increased Hh signaling activity and cell proliferation at E10.5, but not at E11.5 (Figures 9I–K; Supplementary Figure S12F,G).

## Discussion

There are three categories of hearing loss: sensorineural hearing loss, conductive hearing loss, and mixed hearing loss. Primary cilia dysfunction causes a range of diseases known as ciliopathies (Hildebrandt et al., 2011; Mill et al., 2023; Ren et al., 2023). Sensorineural hearing loss as well as conductive hearing loss has been reported in many ciliopathy patients (Hildebrandt et al., 2011; Mill et al., 2023; Ren et al., 2023; Wang et al., 2023; Cappuccio et al., 2022; Kreicher et al., 2018; Tobin and Beales, 2009). We found that *Ofd1* deletion in neural crest-derived cells led to hypoplastic ossicle formation. Similar ossicle phenotypes were observed in mice with conditional deletion of another ciliary protein, *Ift88*, in neural crest-derived cells. These results indicate that primary cilium function is essential for ossicle formation, and dysfunction of primary cilia in neural crest-derived cells results in conductive hearing loss. Thus, *Ofd1* ^*fl*^ *;Wnt1Cre* and *Ift88* ^*fl/fl*^ *;Wnt1Cre* mice are excellent models to understand conductive hearing loss due to hypoplastic ossicle formation. On the other hand, conditional deletion of *Ift88* in the otic epithelium led to sensorineural hearing loss (Moon et al., 2020). Thus, the type of hearing loss is determined by the region in which the primary cilia lose their function. The *OFD1* gene is located on the X-chromosome. One of the copies of the X chromosome is inactivated in female mammals, namely, X-inactivation, which is based on the random choice between the two X-chromosomes. Therefore, in case of the *Ofd1* mutation, the region with *Ofd1* deletion was randomly determined in OFD1 patients. Thus, severities and the type of hearing loss are determined by X-inactivation in Ofd1 syndrome.

Ossicle formation was not initiated, when Hh signaling was absent at E10.5 in *Smo* ^*fl/fl*^ *;Wnt1Cre* mice (Ankamreddy et al., 2019). In addition, our results suggest that many mesenchymal cells lost their identity as ossicular primordial cells, when Hh signaling was downregulated at E11.5. Thus, Hh signaling is required to initiate and maintain ossicular primordial cells in ossicle formation at each stage (Guasto and Cormier-Daire, 2021).

No changes in Hh signaling and primary cilium formation in *Ofd1* ^*fl*^ *;Wnt1Cre* mice at E10.5 indicate that *Ofd1* is dispensable for Hh signaling activation and primary cilium formation at this stage. In fact, the levels of *Ift88* and *Ofd1* expressions in ossicle primordia at E10.5 were significantly lower than those at E11.5 in wild-type mice. Similar findings have been shown in *Ofd*1-mutant limb primordia (Bimonte et al., 2011). Further investigation is required to understand how Hh signaling activation and primary cilium formation occur without *Ofd1* or *Ift88* at E10.5.

We found a lack of primary cilia in *Ofd1* ^*fl*^ *;Wnt1Cre* mice, which was also observed in *Ofd1* ^*fl*^ *;Wnt1Cre;SmoM2* ^*fl*^ mice at E11.5. Hh signaling was not upregulated in *Ofd1* ^*fl*^ *;Wnt1Cre;SmoM2* ^*fl*^ mice at E11.5, when primary cilia were absent in these mice, suggesting that overactivation of *Smo* could not induce primary cilium formation. Furthermore, Hh signaling could not be upregulated by the overexpression of Smo, when primary cilia were absent.

Fetal therapy is defined as a therapeutic intervention, either invasive or noninvasive, for correcting or treating fetal malformations or abnormal conditions (Lin et al., 2021; Waddington et al., 2024). Recently, many fetal conditions have been treated successfully using noninvasive fetal therapy. In these therapies, mothers are treated with medications which are transferred to fetus through the placenta and exert a positive effect on the fetus (Sharma and Tsibizova, 2022a; Sharma and Tsibizova, 2022b). It is known that craniofacial anomalies account for approximately one-third of all birth defects. X-linked hypohidrotic ectodermal dysplasia patients suffer tooth anomalies in the craniofacial region, which were treated with the application of a recombinant protein to the mother, as noninvasive fetal therapy (Schneider et al., 2018). Craniofacial anomalies in Treacher Collins syndrome have also been shown to be rescued by administering antioxidants to the mother mouse (Sakai et al., 2016). Our results indicated that cell proliferation of the ossicular primordium is under control of Hh signaling at E10.5, and it could be increased by the application of Hh signaling agonists to the mother mouse. It is possible that familial hearing loss due to hypoplasia of ossicles could be rescued by increased cell proliferation with agonist application. On the other hand, our data indicate that the efficacy of agonist treatment depends on the presence of intact cilia. Therefore, understanding the timing and location of ciliogenesis defects in human ciliopathy patient must be crucial for developing future treatment strategies to promote proliferation. Alternative approaches should be considered, if ciliogenesis defects persist throughout embryogenesis in patients. Thus, our findings provide hints for possible future treatment for familial hearing loss caused by ossicular hypoplasia.

### Experimental procedures

#### Production and analysis of transgenic mice

All the experimental procedures involving animals were reviewed and approved by the Niigata University Institutional Animal Care and Use Committee (approval number SA00551). *Ofd1* ^*fl/fl*^, *Ift88* ^*fl/fl*^, *Smo* ^*fl/fl*^, *Wnt1Cre*, and *R26SmoM2* ^*fl*^ mice were produced as described by Ferrante et al. (2006), Haycraft et al. (2007), Danielian et al. (1998), and Jeong et al. (2004), respectively. Embryonic day 0 (E0) was taken to be midnight prior to finding a vaginal plug.

### *In situ* hybridization

*In situ* hybridization was carried out to detect mRNAs using [^35^S]UTP, as described previously (Ohazama et al., 2008).

### Immunohistochemistry

Sections were incubated at 4 °C overnight with antibodies to Sox9 (Merck Millipore, Darmstadt, Germany), γ-tubulin (Sigma Aldrich, St Louis, MO, United States), and acetylated α-tubulin (Sigma Aldrich, St Louis, MO, United States). Sections were then incubated with appropriate secondary antibodies. The TSA fluorescein System (Perkin Elmer, Waltham, MA, United States) was used for detecting Sox9. Nuclei were stained with DAPI. The percentage of ciliated cells and the average cilia length were automatically quantified with CiliaQ on ImageJ.

### Three-dimensional reconstruction

Three-dimensional reconstructions were created using Amira software from serial tissue sections stained with hematoxylin and eosin. The volumes of the malleus and incus were subsequently measured.

## EdU

Cell proliferation ability was examined using the Click-iTTM EdU imaging detection kit according to the manufacturer’s instructions (Sigma). EdU is a thymidine analog that can be incorporated to label cells undergoing DNA replication. EdU-positive cells are defined as proliferating cells.

### TUNEL assay

The TUNEL assay was performed using an *in situ* apoptosis detection kit (Roche, Basel, Switzerland), according to the manufacturer’s instructions.

#### Skeletal preparation

For skeletal analysis, pups were stained with Alcian blue for identifyinf nonmineralized cartilage and Alizarin Red for bone. In brief, mice tissues were fixed in 100% ethanol and then stained with 0.1% Alizarin Red S (in 95% ethanol), 0.3% Alcian blue (in 70% ethanol), 100% acetic acid, and ethanol for 5 days, followed by alkaline hydrolysis and glycerol clearing.

#### Quantitative-PCR (Q-PCR)

Embryos were frozen and sectioned into 12-μm-thick slices. Then, the sections were mounted on PEN membrane slides, which were stained with toluidine blue. Ossicle regions were dissected using the Laser micro dissection system (Leica Microsystems, Wetzlar, Germany) into a microcentrifuge tube cap placed directly beneath the section. The tube cap was filled with 75 μL of RNAlater (Sigma Aldrich, St Louis, MO, United States). RNA was isolated using an RNeasy Mini Kit (Qiagen, Hilden, Germany). Q-PCR was performed using GoTaq qPCR Master Mix (Promega, Madison, WI, United States) with the carboxy-X-rhodamine (CXR) Dye and Rotor-Gen-Q (Qiagen) detection system. All samples were run in triplicate for each experiment, and relative transcript abundance was normalized to the amount of GAPDH.

### SAG application

Pregnant female mice were injected intraperitoneally with SAG (AdipoGen Life Sciences, Liestal, Switzerland, 10 mg/kg) once at E9.5.

### Statistical analysis

Excel Toukei (ver. 6.0) was used for statistical analysis, which was done with a two-tailed unpaired Student’s t-test. P < 0.05 was considered statistically significant.

## Data Availability

The original contributions presented in the study are included in the article/Supplementary Material further inquiries can be directed to the corresponding author.
