# Supplementary figures and images for: Early increased cell proliferation compensates subsequent hypoplasia of the ossicle
Source: Front Cell Dev Biol. 2025 Oct 29;13:1627730. doi: 10.3389/fcell.2025.1627730 (PMC12605138; doi:10.3389/fcell.2025.1627730)

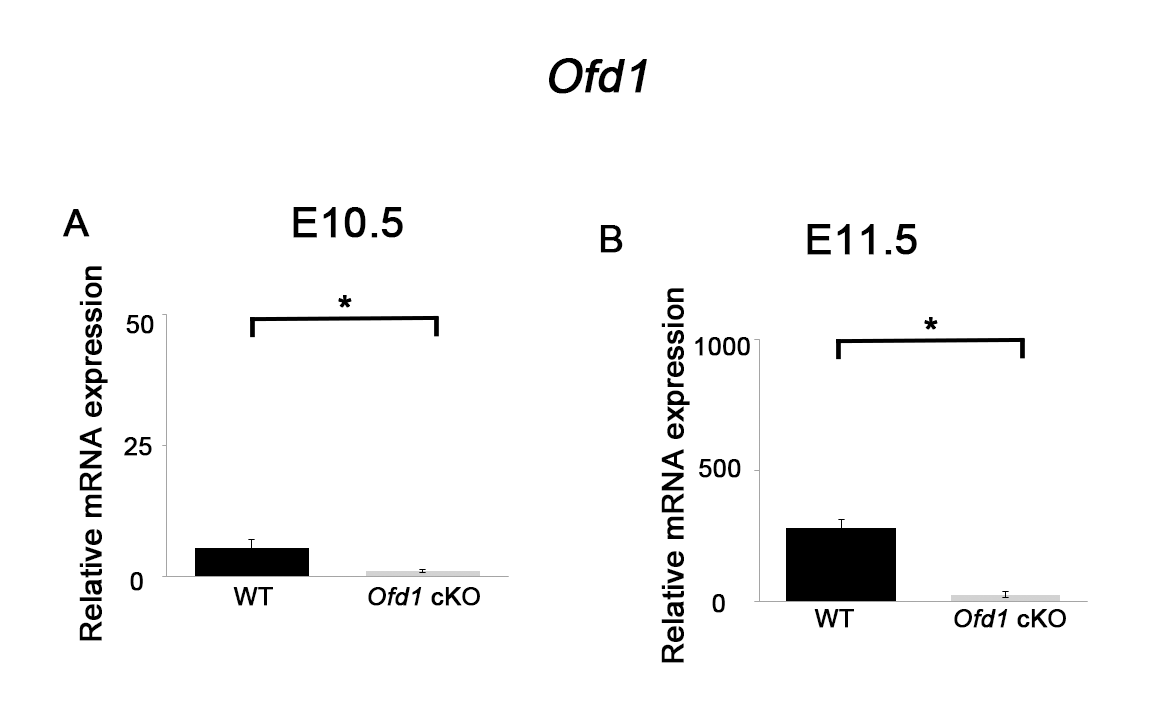

Supplement: Supplementary file 1 [file Image6.tif]

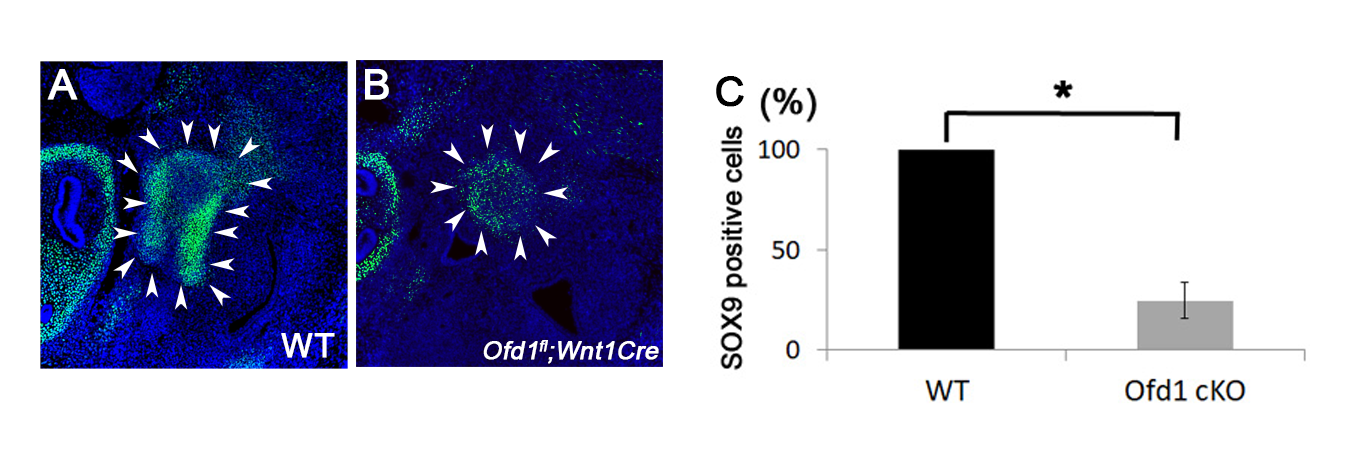

Supplement: Supplementary file 2 [file Image3.tif]

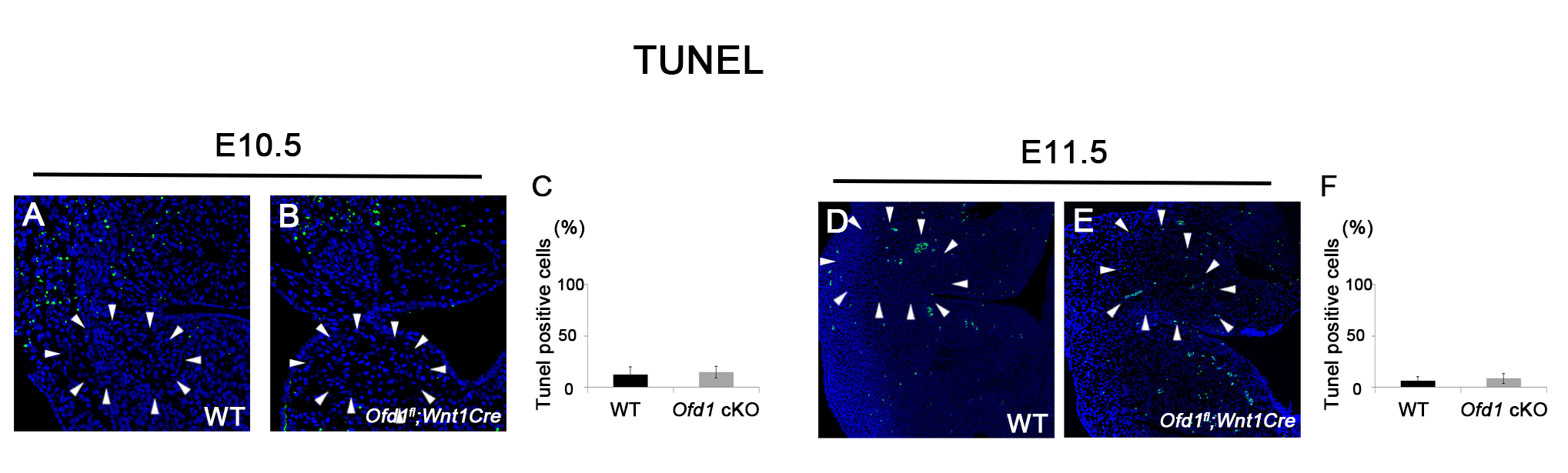

Supplement: Supplementary file 3 [file Image4.tif]

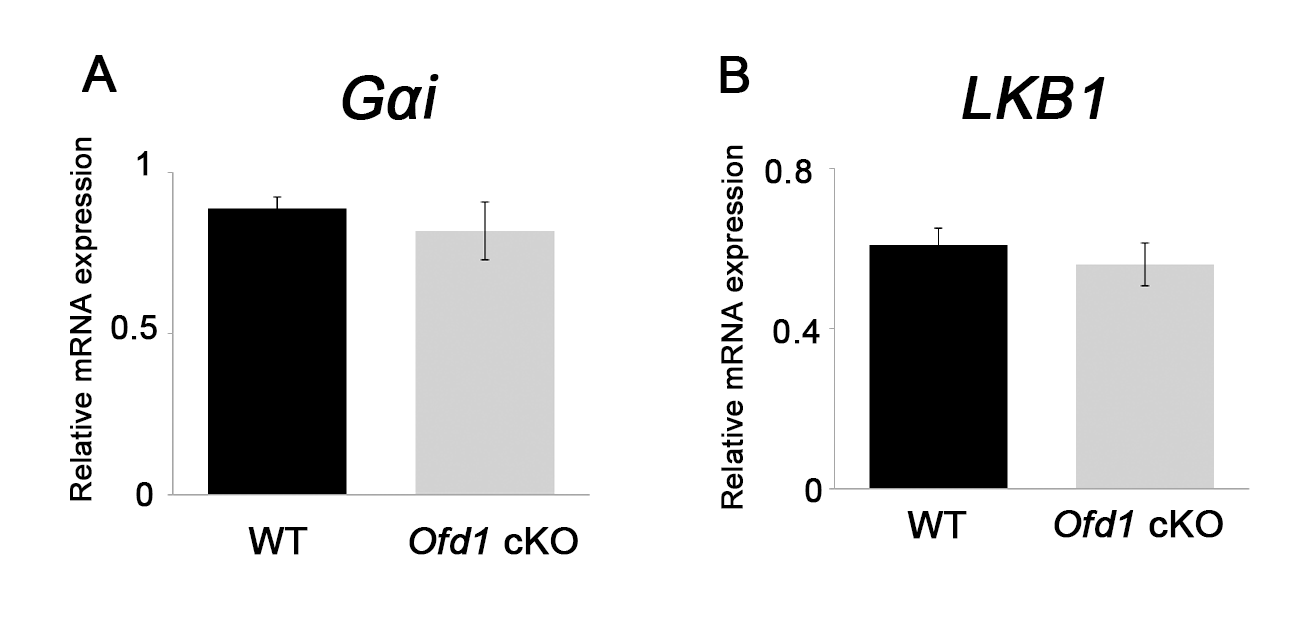

Supplement: Supplementary file 4 [file Image9.tif]

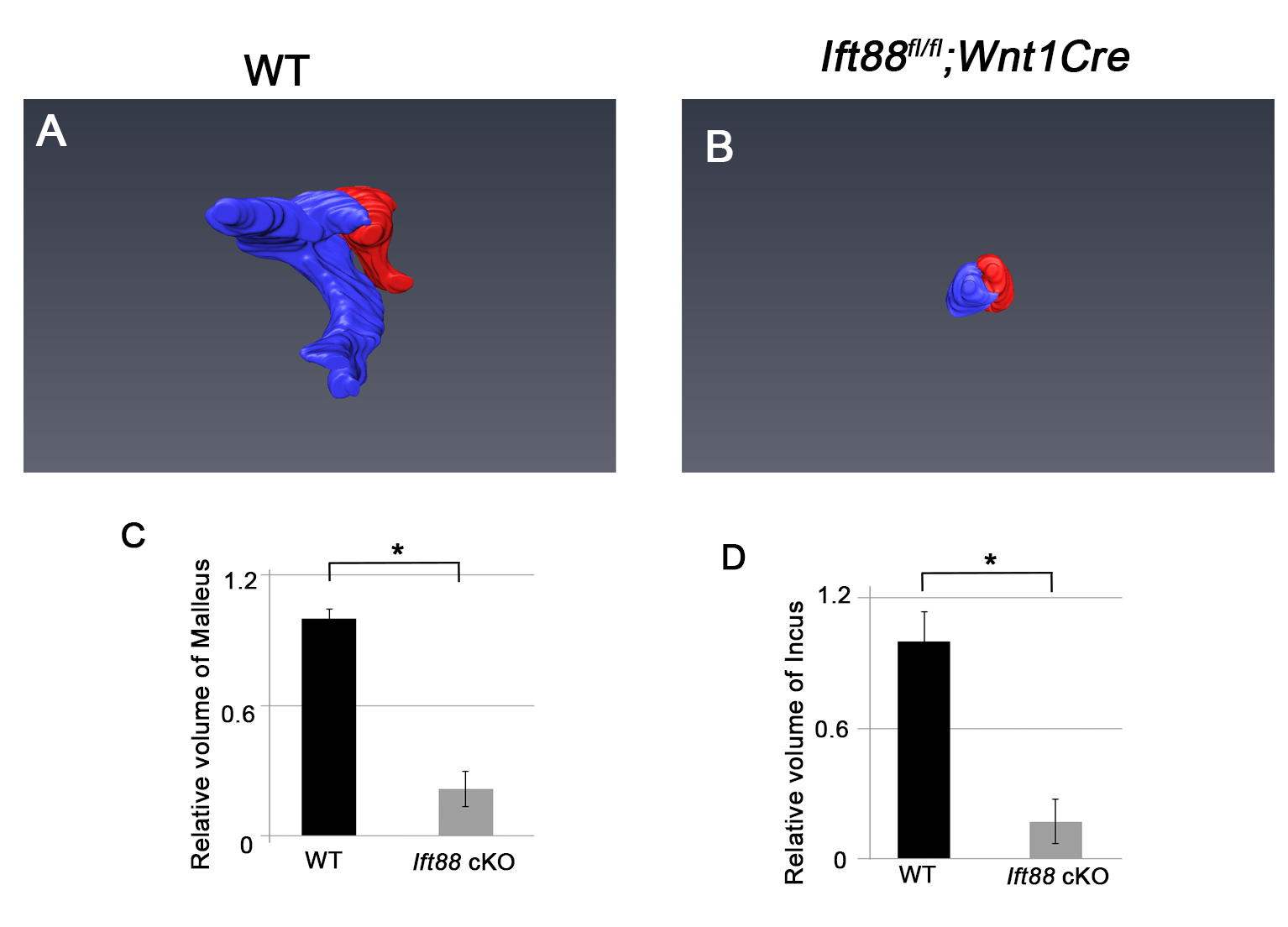

Supplement: Supplementary file 5 [file Image2.tif]

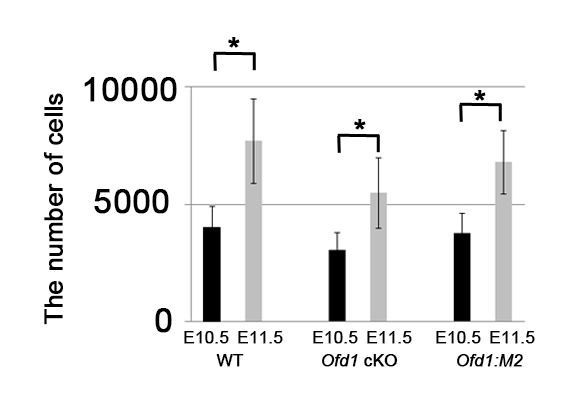

Supplement: Supplementary file 6 [file Image11.tif]

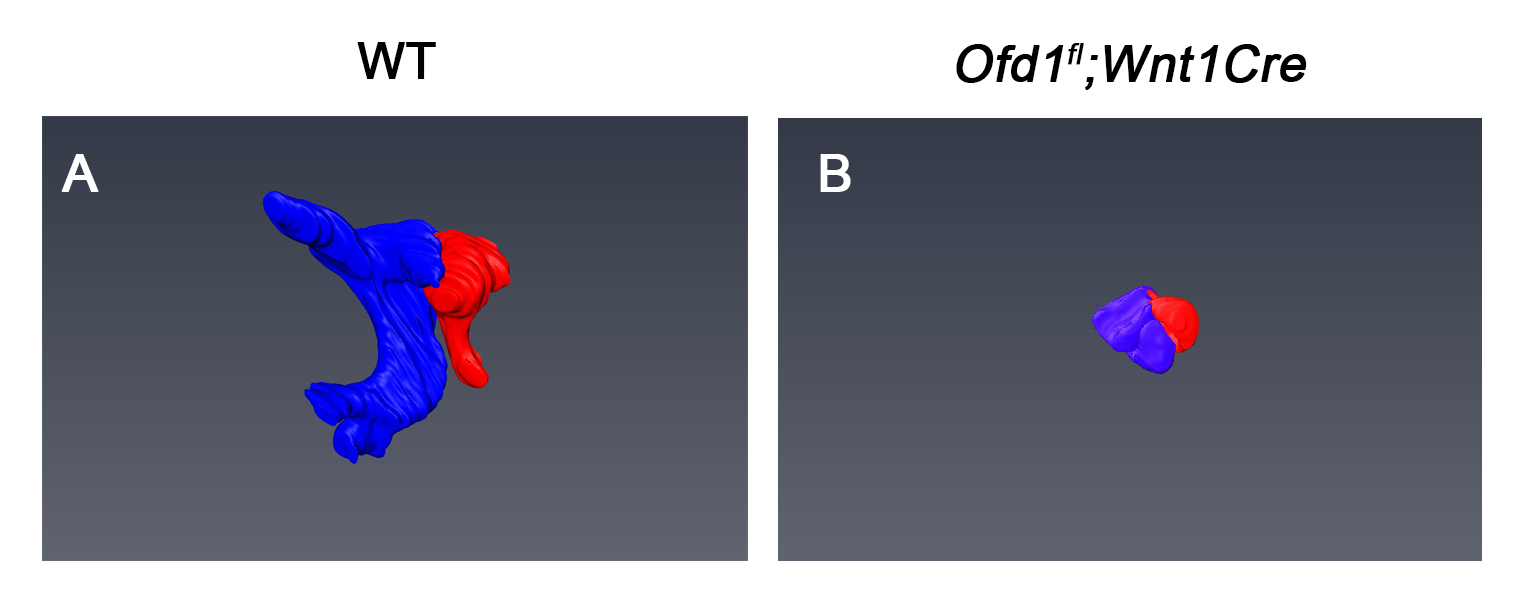

Supplement: Supplementary file 7 [file Image1.tif]

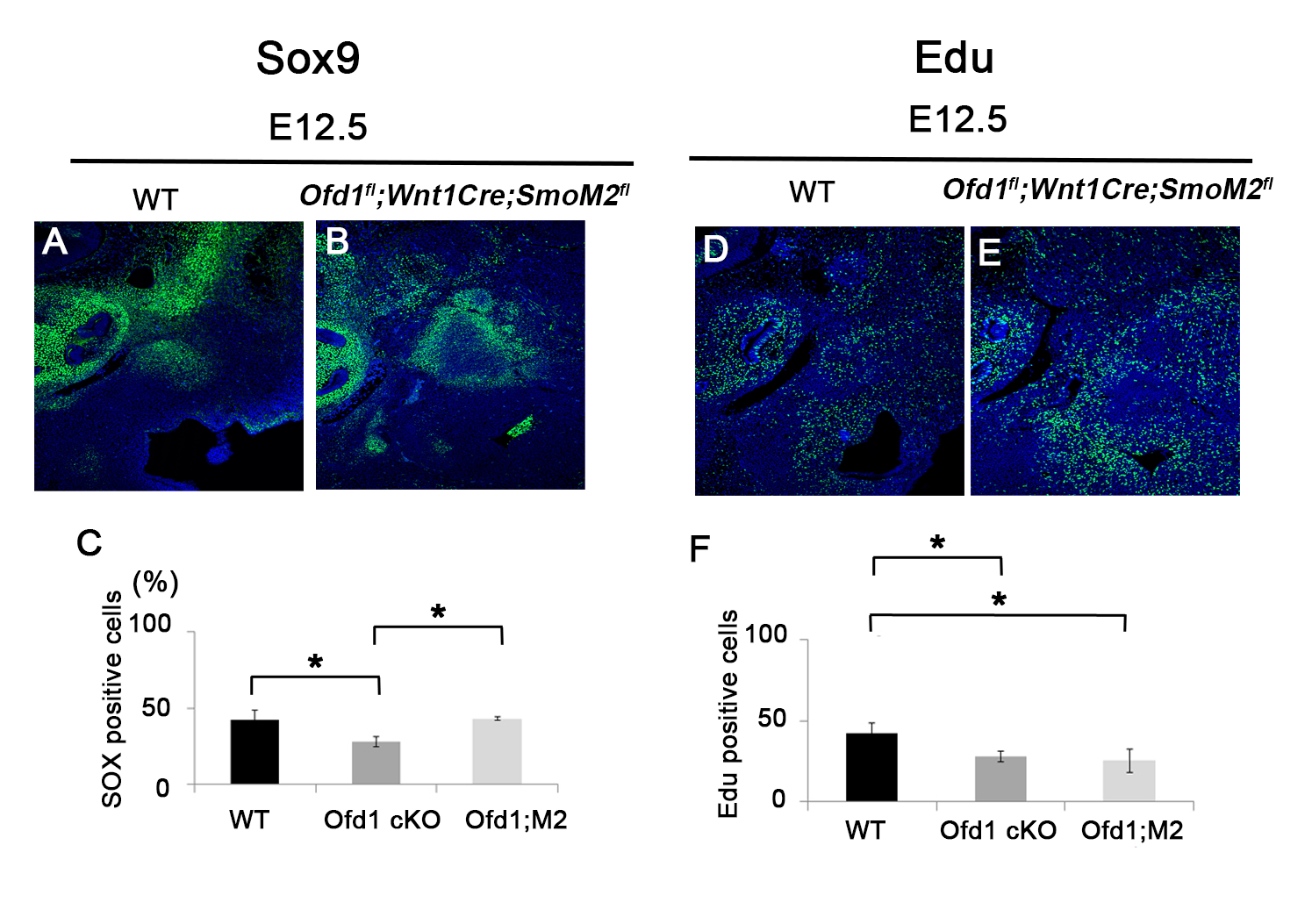

Supplement: Supplementary file 8 [file Image10.tif]

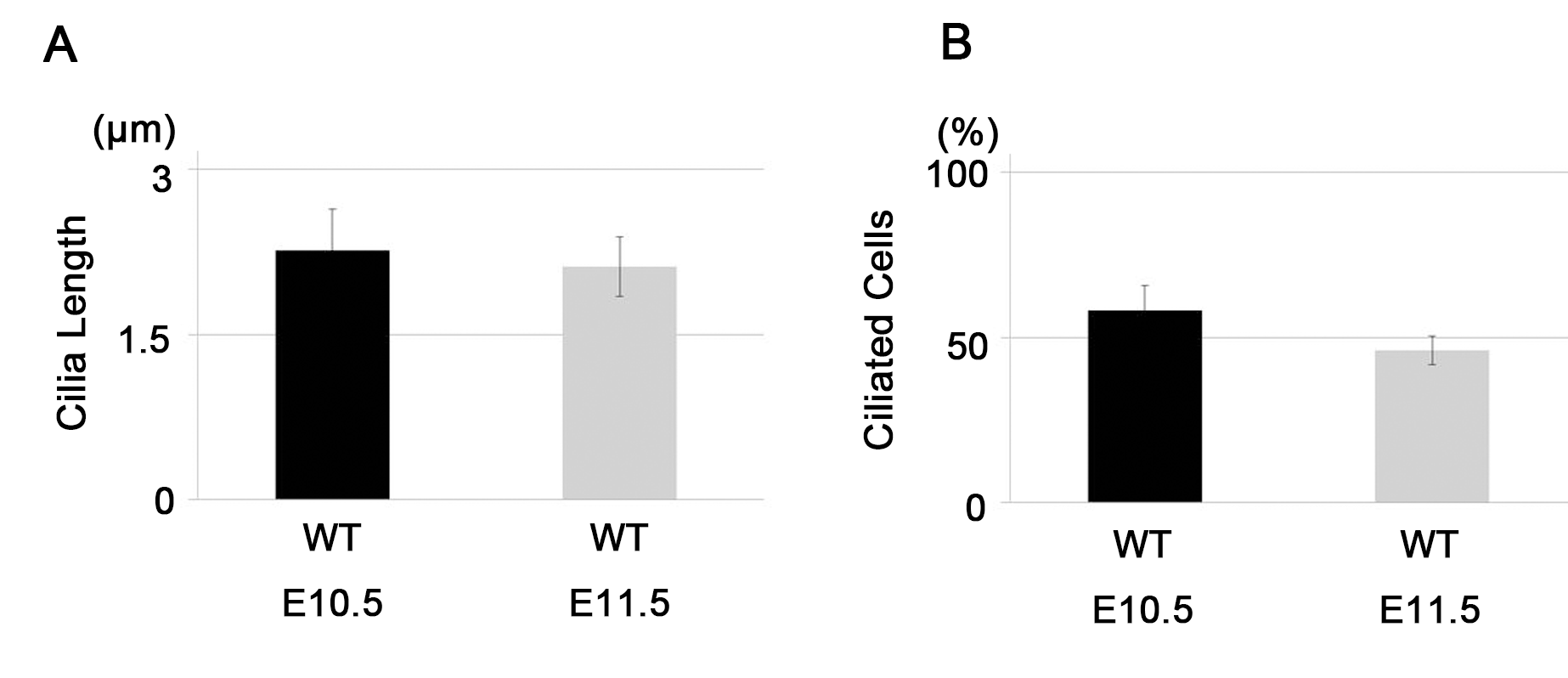

Supplement: Supplementary file 9 [file Image7.tif]

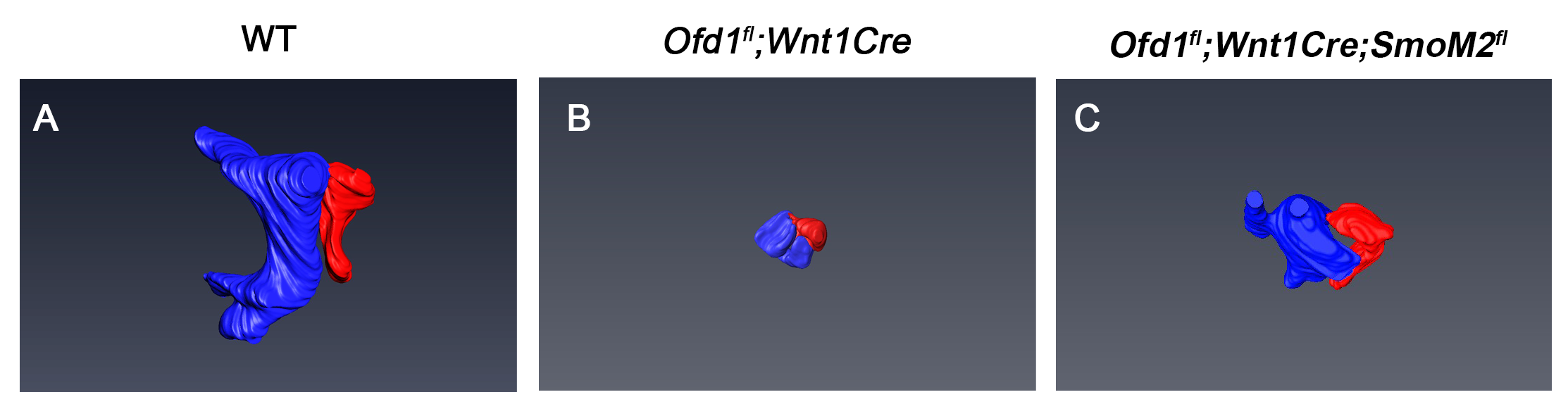

Supplement: Supplementary file 10 [file Image8.tif]

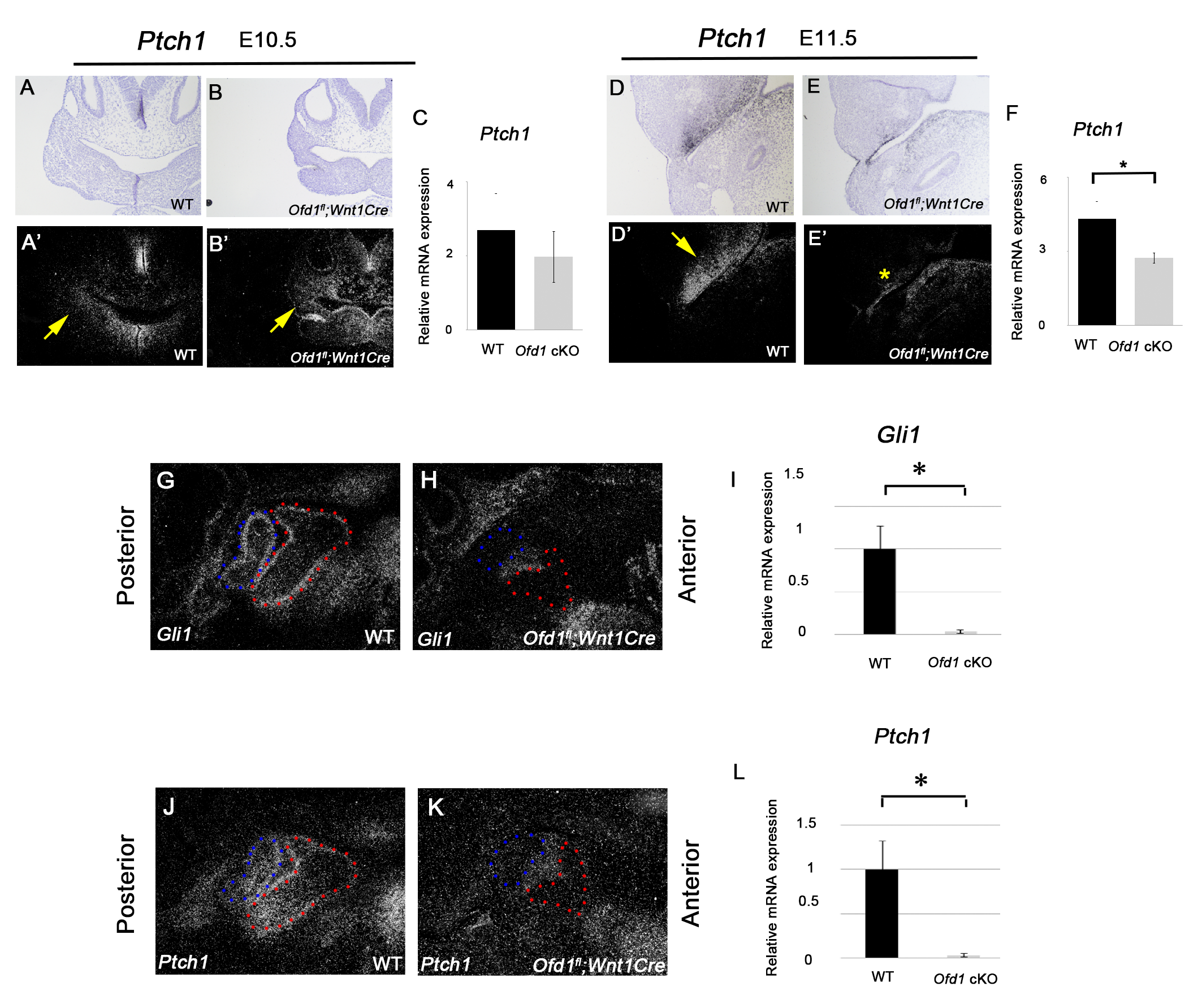

Supplement: Supplementary file 11 [file Image5.tif]

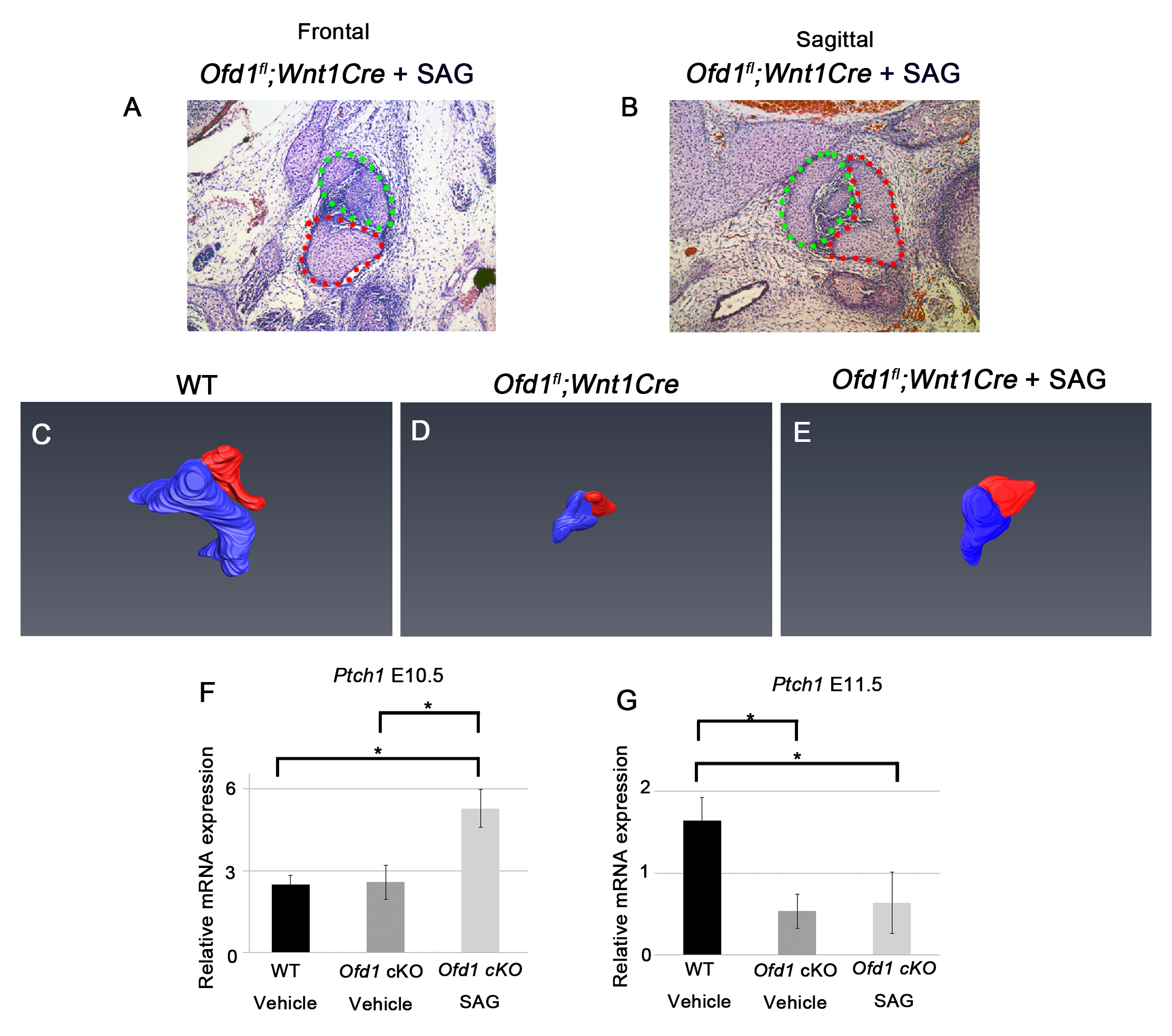

Supplement: Supplementary file 12 [file Image12.tif]
